# Gaps and roadmap of novel neuromodulation targets for treatment of gait in Parkinson’s disease

**DOI:** 10.1038/s41531-021-00276-6

**Published:** 2022-01-11

**Authors:** Rubens Gisbert Cury, Nicola Pavese, Tipu Z. Aziz, Joachim K. Krauss, Elena Moro, the Neuromodulation of Gait Study Group from Movement Disorders Society

**Affiliations:** 1 Movement Disorders Center, Department of Neurology, School of Medicine, University of São Paulo, São Paulo, Brazil; 2 Clinical Ageing Research Unit, Newcastle University, Newcastle upon Tyne, NE4 5PL UK; 3 Nuffield Department of Clinical Neurosciences, John Radcliffe Hospital, University of Oxford, Oxford, UK; 4 Department of Neurosurgery, Hannover Medical School, Hannover, Germany; 5 Center for Systems Neuroscience, Hannover, Germany; 6 Division of Neurology, Grenoble Institute of Neurosciences, Grenoble Alpes University, CHU of Grenoble, Grenoble, France; 7 INSERM U1216, Grenoble Institute of Neurosciences, Grenoble, France

**Keywords:** Parkinson's disease, Parkinson's disease

## Abstract

Gait issues in Parkinson’s disease (PD) are common and can be highly disabling. Although levodopa and deep brain stimulation (DBS) of the subthalamic nucleus and the globus pallidus internus have been established therapies for addressing the motor symptoms of PD, their effects on gait are less predictable and not well sustained with disease progression. Given the high prevalence of gait impairment in PD and the limitations in currently approved therapies, there has been considerable interest in alternative neuromodulation targets and techniques. These have included DBS of pedunculopontine nucleus and substantia nigra pars reticulata, spinal cord stimulation, non-invasive modulation of cortical regions and, more recently, vagus nerve stimulation. However, successes and failures have also emerged with these approaches. Current gaps and controversies are related to patient selection, optimal electrode placement within the target, placebo effects and the optimal programming parameters. Additionally, recent advances in pathophysiology of oscillation dynamics have driven new models of closed-loop DBS systems that may or may not be applicable to gait issues. Our aim is to describe approaches, especially neuromodulation procedures, and emerging challenges to address PD gait issues beyond subthalamic nucleus and the globus pallidus internus stimulation.

## Scope of the problem

Gait and balance impairments are very common in Parkinson’s disease (PD), being major contributors to decreased mobility and quality of life during the disease course^1^. About fifteen years after disease onset, 81% of PD patients experience dopamine non-responsive axial problems, including several types of gait disturbance, postural instability, and frequent falls^1^. In particular, freezing of gait (FoG) highly impairs mobility, affecting 7% of patients in early PD, and around 60% of patients in the advanced stages^2^.

Gait and balance functions are orchestrated by the complex interaction of several neural networks (nodes), including the cerebellar-brainstem-striatal-cortical systems^1^. Information from this circuitry ultimately modulates the final executor: the muscle. Neuronal impairment into this circuity culminates in gait problems, including FoG^3^.

Although dopaminergic medications and deep brain stimulation (DBS) of the subthalamic nucleus (STN) and the globus pallidus internus (GPi) significantly ameliorate cardinal motor symptoms in PD, their effects on gait and balance are less predictable and not well sustained in the long-term^3^. Levodopa has been considered a double-edged sword, improving gait speed and step length as well as turning and arm swing, but also possibly worsening other complex walking skills, such as gait initiation and postural sway^4^. Similarly, DBS improves many of the same parameters as medication, including gait speed and stride length, but with a marginal effect on other gait parameters^5,6^.

Given the high prevalence of gait and balance problems in PD and the limitations of the current approved therapies, researchers have explored alternative brain targets and non-invasive modulation of cortical regions and tried to identify electrophysiological biomarkers of gait impairment to drive stimulation techniques.

The present paper describes the state of the art of novel neuromodulation concepts to treat gait problems in PD, the most recent advances, the uncertainties and the gaps to fill in the field.

## Pedunculopontine nucleus DBS

**Fig. 1:**
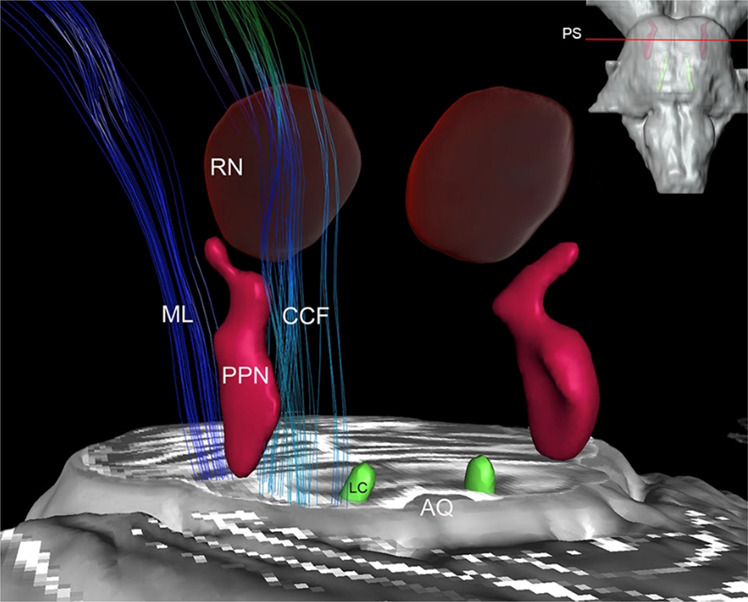
3D reconstruction of the pedunculopontine nucleus. Pedunculopontine nucleus (PPN), red nucleus (RN), locus coeruleus (LC), aqueduct (AQ), medial lemniscus fibers (ML) and cerebellar crossing fibers (CCF) passing around the nuclei. Adapted with permission from Alho et al., 2017.

The PPN is composed by a collection of cholinergic, glutamatergic, and GABAergic neurons with an impressive array of reciprocal connections with basal ganglia, motor cortex, and spinal cord motor neurons^7^. Ascending connections are concentrated mostly on basal ganglia and thalamus, and descending fibers target the spinal cord and the reticular formation^7^. Figure 1 displays a 3D reconstruction of the nuclei^8^.

There has been great hope in PPN area stimulation for gait and balance disturbances in the last two decades^9^. Preclinical DBS experiments^10^ were followed by several clinical trials of PPN area DBS in PD^11^, showing that unilateral or bilateral PPN area DBS could improve gait freezing in both the off- and on-medication states early after surgery (Table 1)^12–14^. However, the degree of improvement has been highly variable and benefits often have not been maintained^15^. PPN area DBS may also have the potential to reduce falls, though the impact on postural instability is unclear^12,15^. Unfortunately, it is unclear whether such partial benefits on gait and balance are clinically meaningful as assessment of quality of life is seldom reported^15^.

**Table 1:** Pedunculopontine stimulation studies in patients with Parkinson´s disease.

Author	Study population	PPN DBS type	Follow-up	Findings
Ferraye et al., 2009	Six PD patients	Bilateral rostral with STN DBS	12 months	Reduced FoG in 4/6 patients. Reduced falls in 1/6 patients.
Moro et al., 2010	Six PD patients	Bilateral caudal lone PPN DBS	12 months	Reduced falls in all patients. Reduced FoG in 5/6 patients at 3 months and 3/6 patients at 12 months.
Thevathasan et al., 2011	Five PD patients	Bilateral caudal lone PPN DBS	24 months	Reduced FoG and falls in all 5 patients at 6 months and 2 years (but lesser benefit at 2 years).
Thevathasan et al., 2012	Seven PD patients	Bilateral caudal lone PPN DBS	2–30 months	Significant improvement in FoG off medication. Bilateral DBS better than unilateral
Welter et al., 2015	Six PD patients	Bilateral rostral and caudal lone PPN DBS	6 months	One patient required device removal due to infection and one patient presented a brainstem bleed. Of the remaining 4 patients: reduced FoG in 3 and reduced falls in 2.
Mestre et al., 2016	Eight PD patients	Unilateral rostral lone PPN DBS	24–48 months	Reduced falls at 2 years in 6/8 patients and at 4 years in 4/6 patients. Reduced FoG at 2 years in 5/8 patients and at 4 years in 4/6 patients.
Perera et al., 2018	Thirteen PD patients	Bilateral caudal lone PPN DBS	6–60 months	Pedunculopontine nucleus stimulation improved intermittent switching of postural sway, feedback gains in the proportional-integral-derivative model and clinical balance impairment.

*PD* Parkinson’s disease, *FoG* freezing of gait, *STN* subthalamic nucleus, *PPN* pedunculopontine nucleus, *DBS* deep brain stimulation.

### Gaps

The clinical application of PPN area DBS is still coupled with several gaps, including patient selection, optimal lead location and stimulation technique^16^. The two “classical” patients’ profiles for PPN area DBS, i.e., (1) PD patients with severe gait freezing resistant to medication or (2) PD patients with resistant gait freezing after STN or GPi DBS, are not related to any predictive factor. A larger sample size and longer outcomes will be particularly important in gauging the success of this intervention. However, refinement of anatomical and physiological data should be first considered.

#### Where is the “sweet spot” for stimulation?

The PPN is embedded in the mesencephalic locomotor region (MLR), together with the cuneiform nucleus and the mesencephalic reticular nucleus. Although the region of the PPN can be visualized in MRI and targeting accuracy has been confirmed on histology^17,18^, the boundaries of the MLR is hard to determine due to the reticular nature of the system and uncertainty of the extension of this physiological system in humans^16^. For example, a study in cats suggests that the cuneiform nucleus is the essential area for stimulation-induced locomotion^19^. Two clinical studies in PD have shown that the best effects on gait occur with active contacts located slightly posterior to the PPN pars compacta, probably in the ventral part of the cuneiform nucleus^12,20^. Consequently, the close vicinity of PPN with other midbrain locomotor structures do not allow us to affirm that the stimulation effects is assigned to a single structure, thus the term “MLR stimulation” may be more appropriate.

Besides doubts about the boundaries of the anatomic target structures, there are divergent opinions on the specific cell type to stimulate. Most studies hypothesize that caudal PPN DBS would be more effective in improving gait problems because it reaches more cholinergic neurons, although this is supported by very limited data^15^. Recent animal studies contradict the original rationale that the PPN cholinergic neurons are critical for gait and put the glutamatergic neurons as the main player in the locomotion activity. Neither indiscriminate excitotoxic lesions of PPN neurons nor selective lesions of cholinergic PPN neurons produced marked abnormalities in gait in rodents^21^. Furthermore, stimulation of glutamatergic PPN neurons in mice (which is also expressed in the caudal region) significantly accelerated locomotion while stimulation of GABAergic PPN neurons slowed locomotion^22,23^.

In summary, (i) beneficial effects of stimulation cannot necessarily be attributed to the PPN itself but may reflect current spread into neighboring structures including the cuneiform nucleus; (ii) the target neuronal population (cell type) to be modulated is still undefined.

### Roadmap

#### Retrospective analysis

Given the successes and failures among different surgical centers, a multicenter analysis of pooled data from PPN DBS patients looking at potential predictors of response related to the precise location of the stimulation, including investigation of the volume of tissue activated and the connectivity patterns with distant areas related to gait mechanism, is crucial^21^.

#### Paradigm of stimulation

Assuming the uncertainties surrounding the nature of the MLR and the neuronal heterogeneity, electric fields generated by DBS electrodes could be fine-tuned with directional steering electrodes or longer linear electrodes to selectively target relevant neurons, including different areas in the rostral caudal axis. Additionally, because cholinergic and glutamatergic neurons are denser in the caudal part and GABAergic neurons show the opposite pattern, different frequencies may improve response to stimulation—possibly by using low frequencies in the caudal part and high frequencies (for inhibition) in the rostral region, through independent current DBS device systems. Noteworthy, a prospective pilot trial of directional cuneiform nucleus DBS is currently underway (clinicaltrials.gov NCT04218526).

Besides different stimulation parameters, DBS electrodes should selectively and precisely stimulate the different intermingled neuronal subpopulations distinguishable by function and neurotransmitter identity in MLR. With that regard, biomarkers to guide DBS protocols could be helpful.

#### Biomarkers

Extracellular recordings of the electrical activity of single or multiple neurons in order to advance our knowledge of the functional disturbances associated with human neural disorders are of utmost importance. Considering that there is no definitive anatomic structure and cell type to target, searching for fingerprints of abnormal electrophysiological activity related to gait is likely to inform closed-loop stimulation. Preliminary studies have shown that alpha-band oscillations in the PPN area are present during rest and while walking and correlate with gait speed^24^. Gait freezing has been associated with attenuation of alpha activity, which begins around 1 s prior to the onset of freezing and continues for over 2 s thereafter^24^. Hypothesizing that the transient reduction in PPN activity during FoG could be a result of excessive inhibition from the GPi and SN pars reticulata, this ‘neuronal signature’ could be the trigger for PPN DBS closed-loop stimulation protocols.

Taken together, deeper circuitry understanding along with electrophysiological data from implanted stimulation electrodes could pave the way for a more effective approach in the future.

## Substantia nigra pars reticulata DBS

Along with the GPi, the substantia nigra pars reticulata (SNr) is a primary output nucleus of the basal ganglia^25^, with GABAergic neurons projecting to the thalamus and the MLR, especially the PPN^26,27^. The pathological overactivity of the SNr observed in PD is thought to lead to inhibition of the locomotor network and is considered one of the major mechanisms responsible for the axial problems in PD^28^. Several studies have emphasized the crucial position of the SNr during locomotion^25,29,30^.

In a rat model of PD, high-frequency SNr stimulation improved forelimb akinesia^31^. In clinical studies, combined stimulation of the SNr (using caudal contacts) and the STN (using rostral contacts) has been attempted to improve locomotion^32,33^. In a double-blind randomized controlled clinical trial, combined stimulation of the STN and the SNr at the same frequency (125 Hz) was superior in controlling resistant FoG compared to STN stimulation alone, whereas balance impairment remained unchanged^32^. A cross-over randomized trial investigated the effects of simultaneous stimulation in both the STN and SNr at different frequencies in PD (126 Hz in STN and 63 Hz in SNr)^34^. This study compared the combined stimulation with the STN or the SNr stimulation alone. For most patients, the combined paradigm yielded the best balance and freezing control, suggesting that the add-on SNr stimulation to STN DBS alone can effectively address PD-associated gait problems^34^.

When directly comparing STN to SNr, an open study showed that STN was superior to SNr for gait and balance control, although SNr stimulation improved the temporal parameters of gait compared to the off condition^35^. Another study showed that stimulation of the SNr but not of the STN was better at controlling anticipatory postural adjustments in PD (Table 2)^30^.

**Table 2:** Substantia Nigra Pars Reticulata stimulation studies in patients with Parkinson´s disease.

Author	Study population	SNr DBS type	Follow-up	Findings
Chastan et al., 2010	Seven PD patients	Bilateral STN stimulation with one contact of each electrode located within the SNr	43 months	Bilateral SNr stimulation improved axial motor symptoms (gait and balance disorders) but had no effect on distal Parkinsonian motor symptoms.
Weiss et al., 2013	Twelve PD patients	Bilateral STN stimulation with caudal contacts stimulating the SNr (interleaving stimulation)	31 months	Combined stimulation of the STN and the SNr at the same frequency (125 Hz) was superior in controlling resistant FoG compared to STN stimulation alone, whereas balance impairment remained unchanged between both conditions.
Scholten et al., 2017	Twelve PD patients	Bilateral STN stimulation with caudal contacts stimulating the SNr	34 months	SNr stimulation improved temporal parameters of gait (swing time asymmetry).
Valldeoriola et al., 2019	Six PD patients	Bilateral STN/SNr stimulation	36 months	Four patients presented the best results with combined stimulation (STN + SNr) while two patients with STN stimulation alone. SNr stimulation alone did not produce better results than combined or STN stimulation alone in any patient.
Heilbronn et al., 2019	Fourteen PD patients	Bilateral STN stimulation with caudal contacts stimulating the SNr	41 months	SNr but not STN stimulation modulated the anterio-posterior size of APA. The SNr modulation of APA was associated with the stimulation effect on FoG

*PD* Parkinson’s disease, *FoG* freezing of gait, *STN* subthalamic nucleus, *SNr* substantia nigra reticulata, *DBS* deep brain stimulation, *APA* anticipatory postural adjustment.

### Gaps

#### What is the best frequency to stimulate the SNr?

The SNr was initially stimulated with conventional DBS devices able to stimulate multiple sites at the same frequency. To control parkinsonian symptoms, high frequency was used on both STN and SN contacts^32^. Subsequent studies using different devices suggested that stimulating the STN and SNr at different frequencies (high and low, respectively), may be more efficacious. This is still controversial and no comparative data between low- and high-frequency SNr stimulation is available.

In parkinsonian rats, SNr stimulation at 150 Hz improved forelimb akinesia and decreased beta oscillations (12–30 Hz) in the SNr as well as SNr neuronal spiking activity. Also, neuronal spiking activity was increased in the ventromedial thalamus, the primary SNr efferent^31^. Conversely, stimulation at 50 Hz did not improve akinesia or beta oscillations in the SNr. This animal data was not confirmed in humans. A cell firing recording during stimulation trains in PD patients across a range of frequencies (1–100 Hz) showed that STN firing attenuated with 20 Hz, and was silenced at 100 Hz, while SNr activity decreased at 3 Hz, and was silenced at 50 Hz. These finding suggest that SNr can be effectively stimulated at lower frequencies (and lower than STN) and that both frequencies used in the available clinical trials (63 Hz or 125 Hz) should be effective rather than exhibit distinct neurophysiological effects. It is conceivable that high frequency inhibits the GABAergic projections directed from SNr to the MLR, but what high frequency means to SNr is still unclear.

#### Electrode positioning is undefined

The location of the DBS electrode within the SNr may play a crucial role in effective treatment^36^. Few preclinical studies suggest that stimulation in the lateral SNr is less effective for treating gait disturbances in PD than stimulation in the medial SNr region^36^. On the other hand, stimulation of the medial portion of the SNr has been shown to induce depression^37^ and hypomania^37^, probably because the medial SNr receives input from nonmotor portions of the ventromedial STN. Consistent clinical studies are lacking.

### Roadmap

The SNr is by far the least-studied target for gait in PD. Studies compiling more patients with longer follow-up data are needed. Of clinical relevance, further work should assess the SNr DBS effects on the spatial and temporal parameters of gait and balance and potential nonmotor complications such as hypomania. Analysis of the volume of tissue activated for best motor outcome together with chronic neuronal recording within the SNr subregions would help to optimize electrode positioning.

Previous work points out that the higher the stimulation frequency, the longer SNr inhibition is achieved^29^; however, how different frequencies directly affect gait parameters need to be systematically compared. Finally, the downstream effects of SNr stimulation are not yet understood and might guide basic research protocols.

## Spinal cord stimulation

Spinal cord stimulation (SCS) has been applied for many years in the management of refractory neuropathic pain due to its good efficacy profile and safety^38^. In the last decade, SCS has been suggested to improve locomotion in PD patients^39^. The potential therapeutic application of SCS received considerable interest after a study in rodent models of parkinsonism demonstrated that stimulation at the thoracic level could improve locomotion^39^.

However, the first clinical study investigating the effect of cervical SCS on motor function in two patients with PD failed to show any benefit^40^. An open-label study including 15 PD patients with low back and/or lower limb pain and thoracic SCS reported a significant improvement in pain intensity, postural stability, and gait speed over 12 months of follow-up^40^. Another open-label study reported improvements in several gait parameters after thoracic SCS for six months in five PD patients^41^. More recently, an open-label study with 6 pain-free PD patients failed to show any benefit 12 months after thoracic SCS^42^. The most relevant studies are summarized in Table 3.

**Table 3:** Spinal cord stimulation studies in patients with Parkinson´s disease.

Author	Study population	SCS level	Follow-up	Findings
Thevathasan et al., 2010	Two PD patients with advanced disease	Cervical SCS (Level C2) at 130–300 Hz; 240– 200 μsec	10 days	There were no differences in gait function (10 m walk)
Fénelon et al., 2012	One PD patient with failed back surgery syndrome	Thoracic SCS (Level T9–10) at 100–130 Hz; 410 μsec	29 months	The motor score and subscores of UPDRS-III were reduced by 50% on average when SCS was switched on in off-drug condition. Waking time was reduced by 21%.
Agari and Date 2012	Fifteen PD patients with low back and/or lower limb pain. Seven patients had DBS.	Thoracic SCS (Level T7–12) at 5–20 Hz, 210–330 μsec	12 months	Patients showed significant improvement in pain intensity, postural stability and gait (timed up and go and 10-m walk) at 3 months and 1 year after surgery.
Landi et al., 2012	One PD patient with DBS and lower limb pain	Thoracic SCS (Level T9–10) at 30 Hz, 250 μsec	16 months	Patient showed significant improvement in pain intensity and tome to walk 20-m (reduced by 20%). The UPDRS-III did not change. Quality of life improved by 60%.
Hassan et al., 2013	One PD patient with refractory neck and upper limb pain	Cervical SCS (Level C2) at 40 Hz; 500 μsec	24 months	Patients showed significant improvement in pain intensity, UPDRS-III (reduced by 41%) and 10-m walk test (reduced by 35%) after 2 years.
Nishioka and Nakajima, 2015	Three PD patients with refractory low back and lower limb pain	Thoracic and lumbar SCS (Level T8–L1) at 5–65 Hz; 420–450 μsec	12 months	Patients showed significant improvement in pain intensity, UPDRS-III scores including rigidity and tremor. Gait was not assessed.
Pinto de Souza et al., 2017	Four PD patients with gait disturbances previously treated with DBS	Thoracic SCS (Level T2–4) at 300 Hz; 90 μsec.	6 months	Patients had ~50–65% improvement in gait measurements and 35–45% in UPDRS III and quality-of-life scores. To analyze placebo effect, blinded SCS was delivered at either 60 or 300 Hz; despite similar paresthesia, gait improvement was only observed with 300 Hz.
Akiyama et al., 2017	One PD patient with advanced disease and DBS with painful camptocormia with Pisa syndrome	Thoracic SCS (Level T8) at Program 1: 7 Hz, 450 μsec Program 2: 7 Hz, 250 μsec	1 month	Patients showed significant improvement in pain intensity, UPDRS-II (reduced by 29%) and timed up and go (reduced by 53%). Camptocormia also improved observed by angles of forward flexion from the vertical axis.
Kobayashi et al., 2018	One PD patient with refractory low back	Thoracic SCS (Level T6–8) at Burst stimulation (inter-burst rate: 40 Hz, intra-burst rate: 500 Hz); 1000 μsec	2 weeks	BurstDR stimulation improved back pain, gait speed and the stooping posture. The UPDRS-III reduced by 70%.
Samotus et al., 2018	Five PD patients with gait disturbances and freezing of gait	Thoracic SCS (Level T8–10) 30–130 Hz; 300–400 μsec	6 months	Mean step length, stride velocity, and sit-to-stand improved by 38.8%, 42.3%, and 50.3%, respectively, Mean UPDRS, Freezing of Gait Questionnaire, and activities-specific balance confidence scale scores improved by 33.5%, 26.8%, and 71.4%, respectively.
Mazzone et al., 2019	Eighteen patients with PD or atypical parkinsonism; patients with and without back pain. Three patients had DBS.	Cervical SCS (Level C2–3) at Tonic (135–185 Hz; 60–210 μsec) or Burst (inter-burst rate: 40 Hz, intra-burst rate: 500 Hz) stimulation	12 months	Both stimulation protocols improved the outcomes. Burst was more effective than tonic stimulation in reducing pain, UPDRS scores and gait. A slight decrease of effectiveness for pain and motor control was observed at the last follow-up for both waveforms, but burst mode showed attenuated decrease.
Samotus et al., 2020	Four PD patients with gait disturbances and freezing of gait	Thoracic SCS (Level T8–10) 30–130 Hz; 300–400 μsec	36 months	Participants demonstrated a reduction in the number of FOG episodes during straight walking at 3-years compared to pre-SCS. Mean FOG-Q and PDQ-8 scores were reduced by 18.3% and by 21.9%; other gait parameters showed great variability between the patients.
Furusawa et al., 2020	Five PD patients with lower back pain	Thoracic SCS (Level T8–10) at Burst stimulation (inter-burst rate: 40 Hz, intra-burst rate: 500 Hz); 1000 μsec	6 months	BurstDR stimulation improved back pain, gait speed and the total UPDRS-III. FOG and tremor scores did not change significantly after SCS.
Prasad et al., 2020	Six PD patients without pain	Thoracic SCS (Level T10)	12 months	There was no clinically meaningful effect on patients’ mobility.
Cury et al., 2020	One PD patient without pain	Thoracic SCS (Level T2–4) at continuous or cycling stimulation (cycling mode: 15 min on-/15 min-off-stimulation)	6 months	Patient did not improve at continuous stimulation but improved the speed and the FOG on the cycling mode

*PD* Parkinson’s disease, *FOG* freezing of gait, *SCS* spinal cord stimulation, *UPDRS-III* Unified Parkinson’s Disease Rating Scale part III, *FOG-Q* freezing of gait questionnaire, *PDQ-8* Parkinson’s Disease Questionnaire.

### Gaps

Despite the overall good outcomes of SCS in treating gait problems in most studies (Table 1), there is still skepticism about the real effects in PD, the protocols to be applied, the long-term effects and the mechanism of action^40^. A relatively small number of PD patients have been evaluated with variable study populations and, so far, no double-blind assessments. Patients are well aware of treatment allocation, and the stimulation produces tangible sensations which might be responsible for a placebo effect. Overall, studies included patients with “gait problems” without specifying which gait patterns and problems were criteria needed for inclusion (e.g., impaired gait velocity, imbalance, freezing of gait, etc..). In addition to the excessive broadness of the inclusion criteria, many papers included patients with lower limb and back pain. This is a confounding bias because pain improvement after SCS can affect gait performance, although clearly identifiable problems such as FoG would be unlikely to be confused with an amelioration of antalgic gait.

#### The stimulation protocol is undefined

The geometry of the stimulation electrodes used in clinical studies has longitudinal current distribution, while a transverse configuration was used in rodents, allowing for coverage of most of the dorsal surface of the spinal cord. Furthermore, few studies have chosen to stimulate the cervical spinal level^40^. Data from SCS used for chronic pain showed that stimulation at a high cervical level preferentially recruits sensory fibers from the upper limbs and chest and rarely recruits fibers from the lower half of the body^43^. However, even in thoracic-level studies, upper and lower levels have been attempted with mixed results (Table 3)^40^.

Besides stimulation levels, there is a high heterogeneity of stimulation parameters with a broad range of frequencies and pulse widths. For example, a study with thoracic SCS randomly delivered either 60 or 300 Hz, and improvements in gait speed were observed only at 300 Hz^44^. In contrast, another study found that lower frequencies (30–130 Hz) benefit PD gait problems^41^.

#### Placebo effect cannot be ruled out

PD signs can improve to a remarkable extent following placebo intervention. This is particularly true of freezing, a phenomenon highly influenced by stress, attention, and environmental distractions. Consequently, it is possible that the paresthesia induced by stimulation would result in a placebo effect.

### Roadmap

A study population with better-defined inclusion criteria, multicenter trials, and long-term follow-up are the next steps to consolidate (or not) SCS as a neuromodulatory tool for gait in PD. Double-blind approaches designed with an amplitude subthreshold for paresthesia, very high frequencies (below the sensory threshold)^45^, or new paradigms such as burst stimulation^46,47^ might certainly guide future trials to avoid placebo effects.

Another roadblock in SCS for PD that should be considered next is the lack of a convincing mechanism of action. It has been reported that SCS may disrupt excessive low-frequency synchronous corticostriatal oscillations in monkeys, leading to the appearance of neuronal activity^48^. In humans, SCS has been reported to improve anticipatory postural adjustment, which is found to be modulated by SMA^49^, but no consistent functional neuroimaging and electrophysiological studies are available. Functional neuroimaging and multisite electrophysiological recordings (through EEG and STN or GPi DBS) will help to better understand remote effects of SCS in PD and its potential influence on the cortico-basal ganglia circuitry.

Finally, predictive factors of benefit when considering an invasive procedure are crucial. Unlike the deeply located basal ganglia and brainstem targets already tested for gait, the spinal cord can be non-invasively modulated through transcranial magnetic stimulation (TMS); this paradigm has recently been applied for PD^50^. Consequently, we wonder whether trans-spinal magnetic stimulation given before surgery would be useful as a predictor of response for epidural SCS.

## Adaptive DBS for gait

New DBS systems operate by adapting the stimulation amplitude in response to an input signal (adaptive DBS, aDBS). The most studied input signal in PD is the beta band frequency oscillation, measured in the STN recording local field potential power^51^. Excessive STN beta activity has been shown to correlate with the severity of akinetic-rigid symptoms whereas beta amplitude suppression through therapeutic levodopa or DBS improves rigidity and bradykinesia, thus supporting the use of the beta band power as a biomarker for the parkinsonian off state^51^.

### Gaps

The use of beta as the input information for gait function in PD is a subject of debate. For instance, the frequency within the beta band range seems to be different between standing and gait^52^. More importantly, the exact behavior of STN neuronal frequencies during different phases of the gait cycle is still largely unknown^52^.

Recent evidence from an STN intraoperative recording study has pointed out that FoG is related to transient increases in pathological beta and theta activity^53^. Interestingly, the pathological activity was already observed in the moments prior to freeze onset. In freely-moving PD subjects, freezers demonstrated longer duration beta bursts than non-freezers during gait^54^.

#### Amplitude or frequency adaptive DBS?

Another debate exists around the best parameters to be adjusted in aDBS. A growing body of literature suggests different functional roles for sub-bands within the beta spectrum^52^. Both high- and low-frequency DBS improved limb bradykinesia by attenuating the sub-band of high-beta oscillations in the STN^55^. However, high-frequency DBS also attenuated oscillations across low beta (11–15 Hz) bands, whereas low-frequency DBS amplified these lower beta frequencies^55^. Because low-beta frequencies have been considered to be non-pathological, it is hypothesized that low-frequency DBS may benefit FOG by enhancing neural coupling in cortico-basal ganglia loops and by selecting better oscillation bands than high frequency. Accordingly, high-beta oscillations have been associated with FoG^53^.

### Roadmap

Lessons learned from epilepsy indicate that the prediction of momentary neural events is not straightforward and offers numerous obstacles. Data-driven real-time constant recording in the STN in a larger cohort might refine the threshold for the magnitude and duration of pre-freeze beta and theta modulation. Because FoG is a transient phenomenon, those dynamic oscillations could drive aDBS, acting like a neuronal defibrillator to reset the abnormal signal just before FoG occurs. In addition, besides adapting the stimulation amplitude in response to an input signal, aDBS devices could be designed to adapt not only the amplitude but also the frequency.

Beyond the STN, PPN closed-loop DBS might be promising, and recent work showed its feasibility in five PD patients with both “on” and “off” medication freezing^56^. The primary outcome variable was met in three subjects who exhibited a greater than 40% improvement in the number of FoG episodes from baseline to 6 months during acute PPN closed-loop. However, the group analysis did not reveal a significant benefit. This study established a DBS paradigm driven by an increase in 1-to 8-Hz power within the PPN. Although preliminary, this pilot study motivates the search for better and consistent neuronal biomarkers during chronic recording.

## Non-invasive vagus nerve stimulation

Non-invasive vagus nerve stimulation (nVNS) is an established neurostimulation therapy used in the treatment of epilepsy, migraine and cluster headache^57^. Recently, its implication on gait function in PD has been studied. An open-label, pilot study has analyzed the effect of single dose nVNS on gait pattern in 19 patients with PD-related disorder (twelve with FoG)^58^. A total of two treatments were applied to the left vagus nerve in the left side of the neck. Assessments were performed just before and 15 min following the application of nVNS. The study demonstrated improvement in spatiotemporal gait parameters following nVNS and included step count, velocity, step length, and stride velocity variability. Video-analysis of the FoG patients showed improvements in the time taken for turning, steps taken for turning, and steps taken for start hesitation. A follow-up crossover randomized controlled study corroborated these initial findings and showed significant improvements in walking speed, stance time and step length comparing active phase (30 days of nVNS stimulation) to sham^59^. Similarly, overall motor function (MDS-UPDRS III) also improved. The average duration of freezing episodes was reduced, but other FoG measures did not change. Moreover, serum tumor necrosis factor (TNF)-α and glutathione levels decreased and brain-derived neurotrophic factor (BDNF) levels increased significantly after treatment with nVNS. The authors propose that the ability of nVNS to reduce pro-inflammatory cytokines and to increase serum BDNF could be a sign of neuroplasticity. The effects observed on anti-oxidant levels might also point to disease-modifying actions. nVNS might activate locus coeruleus neurons, which are thought to degenerate even prior to substantia nigra dopaminergic neurons in PD^60^. Improvement in postural instability and gait in PD is expected if there is direct cortical activation through excitatory neurotransmitters such as noradrenaline^61^. The nucleus basalis of Meynert, which provides extensive cholinergic projections to cerebral cortex, is also in receipt of afferent input from locus coeruleus; cortical cholinergic tone is thus also likely to be enhanced by nVNS and could be responsible for gait changes in these patients^62^. Future studies of nVNS in PD should confirm repeatability, optimize treatment parameters and establish how long treatment effects (and potential neuroprotective effects) of nVNS persist. Larger, multi-centre trials of nVNS in PD are warranted.

## Targeting higher-order posture-gait structures

Above we have discussed subcortical regions for gait modulation which in turn are largely influenced by numerous cerebral pathways involved in movement initiation and somatosensory integration, which regulate ongoing movements for anticipatory or feed-forward adjustments^63^. TMS has been implicated as a potential method of improving motor performance and normalizing cortical excitability in PD^63^. A recent meta-analysis showed that rTMS stimulation improved motor symptoms (using the UPDRS-III as a standard motor outcome) with a mild effect size^64^. Per stimulation site, primary motor cortex had the highest effect magnitudes (measured by standard mean difference), followed by dorsal lateral prefontal cortex and supplementary motor area. Overall, the studies have showed that bradykinesia and axial scores, including gait, were the subscores of UPDRS most improved by TMS. There is also evidence that stimulation of the primary motor cortex can specifically modulate FoG, in particular the primary leg area. In a meta-analysis with 102 patients, rTMS showed a beneficial effect on FoG questionnaire scores in PD patients^65^. However, there were no significant differences in turning steps, turning time, or Timed Up and Go.

### Gaps

These results should be cautiously interpreted based on two major factors: first, there is a large heterogeneity of the protocols employed accounting for different cortical targets and number of sessions applied, which compromises the generalization of the current evidence; for example, in depression most studies have focused on a single recommended site and protocol, producing consistent results. Second, there is still a lack of trials in order to assess the placebo effects of stimulation and the real effects of stimulation in large sample of patients.

### Roadmap

Future studies should be designed to identify the specific gait disturbances that respond well to rTMS therapy. Additionally, it remains to be determined whether the possible positive effects of repetitive TMS can be sustained over time. Prolonged stimulation or combined targets^63^ might be more efficacious but need to be further explored. Another promising approach is the use of TMS prior to a training intervention (e.g., prior to physiotherapy or treadmill)^66^. In these cases, the rationale is to strength the effectiveness of synaptic connections and recruit fibers required to improve performance during a given task such as gait^67^.

#### Decoding cortical gait oscillations

Besides improvements in methodology and well-designed clinical studies with TMS, in-depth study of cortical regions can serve as a valuable source of neurophysiological signatures related to gait, such as those preceding a freezing episode^68^. This information ultimately could serve as input signals for DBS or spinal cord stimulation (brain-spine interface), which in turn would fine-tune the stimulation parameters^68^. Because of its high temporal precision, cortical electrophysiology is of considerable interest for studying gait. The use of information derived from electrocorticography to modulate DBS has been shown to be feasible in PD patients with dyskinesia by decreasing the DBS amplitude when cortical gamma oscillatory activity is high (accompanying dyskinesia) and increased stimulation amplitude when it is low^68^.

The behavior of cortical neuronal activity during gait in PD has been scarcely studied. A study showed increased theta-activity in the frontal midline during freezing episodes in PD^69^. A multisite neurophysiological recording showed that FoG is characterized by the breakdown of cortico-subthalamic nucleus coupling, which is evident at the transition from normal walking to gait freezing^52^. Further studies decoding the cortical brain activity related to normal and abnormal gait functions are a necessary step to build valid brain-computer interfaces/machine learning algorithms capable of generating precise information to closed-loop systems.

## Future perspectives and final remarks

**Fig. 2:**
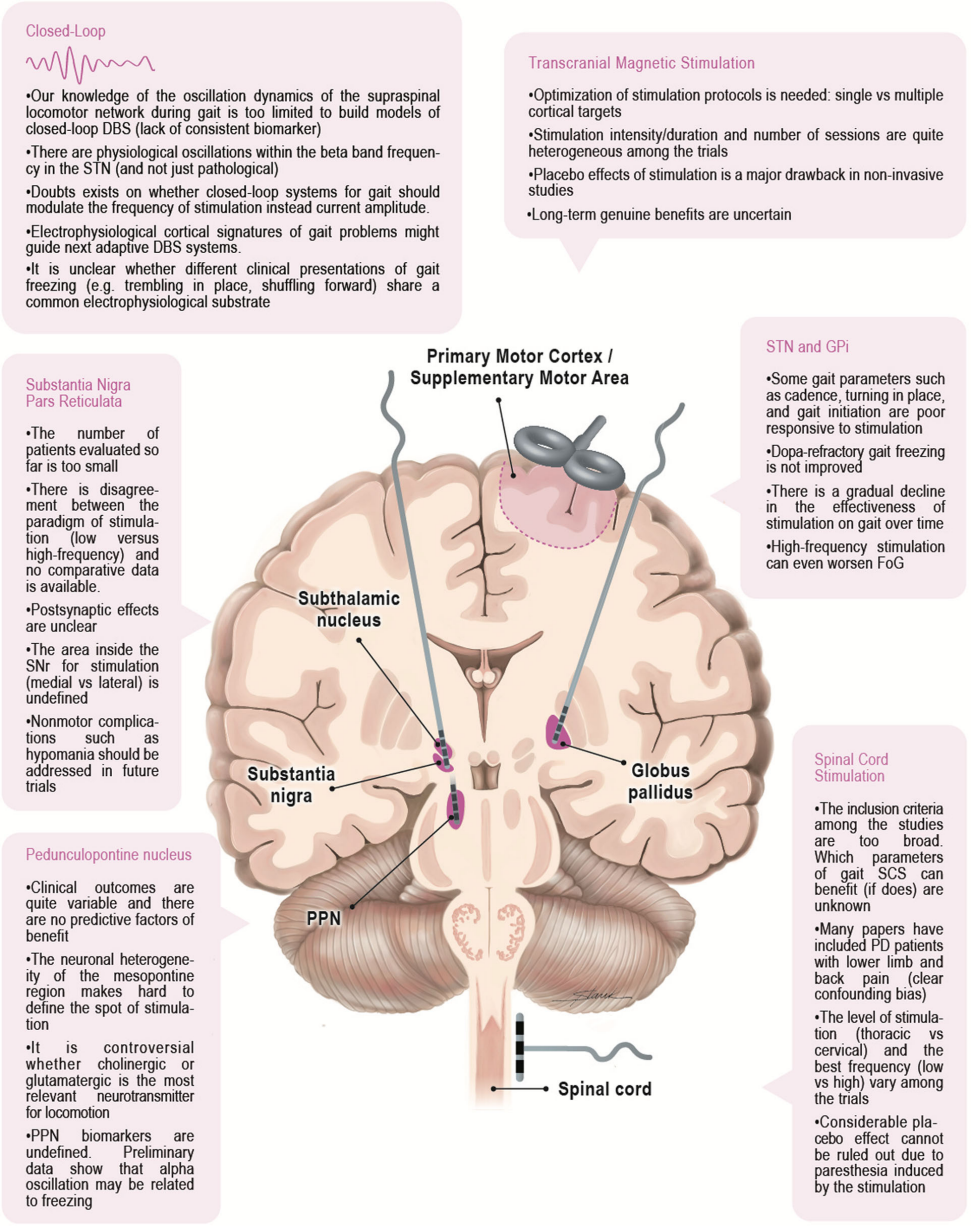
Main gaps for each target and neuromodulation techniques used to treat gait problems in Parkinson’s disease.

The field of neuromodulation for gait in PD has significantly advanced with new targets in the spinal and supraspinal gait network, though outside the conventional targets of STN and GPi. However, there are several critical unanswered questions (summarized in Fig. 2). Much of the very divergent results among the different targets could be related to a lack of a functional-anatomical basis for the different gait abnormalities in PD as approaches to isolate relevant networks are limited. Many of the available studies on locomotion were conducted in rodents and felines, which offer means to circumvent current barriers of studying gait in humans. However, the velocity dependence of gait parameters and differences between quadruped and biped gait have made this comparison challenging^70^. Although it is likely that critical features of locomotion are phylogenetically conserved, the connectivity between nuclei differ between species^70^. Multisite neural recordings and intracranial stimulation are promising tools for evaluating whether it is possible to establish proof of concept for a circuit-targeted precision medicine approach, where dysfunctional neural circuits related to specific gait patterns are reliably identified and targeted^71^. Additionally, further studies of the oscillation dynamics in the locomotor network may prove important to building models of adaptive DBS systems which may or may not be effective. Physiological pathways are intertwined with such conveying pathological activity and thus nonspecific stimulation may result in adverse events and suboptimal outcomes. Biomarkers of abnormal gait states such as FoG may be helpful to detect better pathological processes for modulation of gait. We predict that the road to restoring neural circuit impairment relevant to gait will translate to the use of more specific strategies and to more sophisticated multisite recording utilizing biomarkers.

## Supplementary information

Supplementary Information
