# Supplementary material for: Gaps and roadmap of novel neuromodulation targets for treatment of gait in Parkinson’s disease
Source: NPJ Parkinsons Dis. 2022 Jan 11;8:8. doi: 10.1038/s41531-021-00276-6 (PMC8752758; doi:10.1038/s41531-021-00276-6)
Supplement: Supplementary file 1 — Supplementary Information [file 41531_2021_276_MOESM1_ESM.pdf]

*** Supplementary Information. Neuromodulation of Gait Study Group from Movement Disorders Society**

AlDakheel, Amal; Blahak, Christian; Bloem, Bastiaan R.; Butson, Christopher; Capato, Tamine T.C.; Chabardes, Stephan; Coyne, Terry J.; Debu, Bettina; Grabli, David; Gulberti, Alessandro; Foltynie, Thomas; Fonoff, Eric; Foote, Kelly; Fraix, Valerie; Haas, Aline; Hamani, Clement; Hirsch, Etienne; Horak, Fay; Johnson, Kara; Kalia, Suneil K.; Kang, Un; Kosutzka, Zuzana; Lozano, Andres M.; Mahant, Neil; Okun, Michael; Ostrem, Jill; Prakash, Neha; Potter-Nerger, Monika; Sarangmat, Nagaraja; Silburn, Peter A.; Schrader, Christoph; Stein, John; Stefani, Alessandro; Tai, Chun-Hwei; Tai, Yen; Thevathasan, Wesley; Veletzas, Ioannis; Wagner, Jonas; Welter, Marie-Laure; Zrinzo, Ludvic.
